# Correction to “Seedless One‐Pot Synthesis of Colloidal InAs Quantum Dots Enabling a High‐Accuracy Photoplethysmography Oximeter”

**DOI:** 10.1002/advs.77497

**Published:** 2026-09-29

B. K. Kim, S. Jee, Y. Ahn, et al. “Seedless One‐Pot Synthesis of Colloidal InAs Quantum Dots Enabling a High‐Accuracy Photoplethysmography Oximeter.” *Advanced Science* 13, no. 38 (2026): e24375. https://doi.org/10.1002/advs.202524375

We have just noticed that one of the grant numbers in the Acknowledgements section was entered incorrectly. The grant number was published as RS‐2025‐24563595, whereas the correct number is RS‐2025‐24523595.

Nevertheless, as accurate funding information is important, we would be very grateful if you could kindly consider correcting the Acknowledgements section as follows:

“This work was supported by Korea Research Institute for Defense Technology Planning and Advancement (KRIT)  Grant funded by Defense Acquisition Program Administration (DAPA) (KRIT‐CT‐23‐031); the National Research Foundation of Korea (NRF) grant funded by the Korea government (MSIT, RS‐2025‐24523595, RS‐2025‐02316659); the Korea Research Institute of Standards and Science (KRISS‐JP2026‐0052, KRISS‐GP2026‐0001); and by the Ministry of Trade, Industry and Energy (MOTIE) and Korea Institute for Advancement of Technology (KIAT) through the International Cooperative R&D program (P0028887).”

We apologize for this error.

